# Evaluation of the capacities of mouse TCR profiling from short read RNA-seq data

**DOI:** 10.1371/journal.pone.0207020

**Published:** 2018-11-15

**Authors:** Yu Bai, David Wang, Wentian Li, Ying Huang, Xuan Ye, Janelle Waite, Thomas Barry, Kurt H. Edelmann, Natasha Levenkova, Chunguang Guo, Dimitris Skokos, Yi Wei, Lynn E. Macdonald, Wen Fury

**Affiliations:** 1 Regeneron Pharmaceuticals, Tarrytown, New York, United States of America; 2 Department of Biological Statistics and Computational Biology, Cornell University, Ithaca, New York, United States of America; 3 Robert S. Boas Center for Genomics & Human Genetics, Feinstein Institute for Medical Research, Northwell Health, Manhasset, New York, United States of America; Chang Gung University, TAIWAN

## Abstract

Profiling T cell receptor (TCR) repertoire via short read transcriptome sequencing (RNA-Seq) has a unique advantage of probing simultaneously TCRs and the genome-wide RNA expression of other genes. However, compared to targeted amplicon approaches, the shorter read length is more prone to mapping error. In addition, only a small percentage of the genome-wide reads may cover the TCR loci and thus the repertoire could be significantly under-sampled. Although this approach has been applied in a few studies, the utility of transcriptome sequencing in probing TCR repertoires has not been evaluated extensively. Here we present a systematic assessment of RNA-Seq in TCR profiling. We evaluate the power of both Fluidigm C1 full-length single cell RNA-Seq and bulk RNA-Seq in characterizing the repertoires of different diversities under either naïve conditions or after immunogenic challenges. Standard read length and sequencing coverage were employed so that the evaluation was conducted in accord with the current RNA-Seq practices. Despite high sequencing depth in bulk RNA-Seq, we encountered difficulty quantifying TCRs with low transcript abundance (<1%). Nevertheless, top enriched TCRs with an abundance of 1–3% or higher can be faithfully detected and quantified. When top TCR sequences are of interest and transcriptome sequencing is available, it is worthwhile to conduct a TCR profiling using the RNA-Seq data.

## Introduction

T-cell receptors (TCR), usually consisting of disulfide-bound α and β chains, are expressed on the surface of T lymphocytes and play a vital role in antigen-induced T cell immunity [1]. A large repertoire of diverse TCRs enables T cells to recognize a wide variety of antigens displayed by major histocompatibility complex (MHC) molecules. Upon antigen recognition, TCRs promote a series of signaling cascades that regulate T cell activation, cytokine production, survival and proliferation [2, 3]. Subsequently, clonal expansion of reactive T cells may occur and reshape the repertoire [4]. As a key component of T cell specificity, the TCR repertoire has been actively studied in infection, autoimmunity and immunoncology [5–11]. For example, specific TCR sequences have been identified in patients with Celiac disease, which render T cells reactive to Gliadin [12, 13]. Knowledge of these sequences will allow monitoring of flares as well as response to therapies. Another example is the potential correlation between higher numbers of expanded and/or contracted clones and better response to immune checkpoint blockade in cancer patients [14, 15].

The vast number of possible complementarity determining region (CDR) sequences, along with αβrandom and palindromic nucleotide additions, gives rise to highly diverse TCRs. For example, 10^15^−10^20^ human TCR clonotypes (a clonotype refers to a unique pair of TCR α and β sequences) are theoretically plausible, though the actual number is smaller (10^7^−10^13^) [16–19]. In mouse, the number of clonotypes is estimated to be 10^6^ [20]. As a result, fully probing the highly diverse TCR repertoire requires massive sampling. Established technologies typically employ target-specific PCR amplification of the TCR alpha and beta loci (e.g. 5’ RACE, ARM-PCR, Multiplex PCR), followed by high throughput sequencing (e.g. Roche 454, Illumina Miseq) [21, 22]. These platforms typically offer a sequencing depth of millions of reads at each locus. At such coverage, 10^4^−10^6^ unique TCR alpha or beta sequences may be detected per sample [23–25]. These platforms usually sequence the alpha and beta chains independently. Nevertheless, an accurate pairing of alpha and beta chains has been achieved with a few multiplex PCR-based approaches, either in conjunction with single cell sorting [26–28] or with bioinformatics algorithms [29]. These breakthroughs further empower the targeted repertoire sequencing approaches.

On the other hand, determining TCR repertoire from whole transcriptome sequencing (RNA-Seq) has gained attention [30–36]. The main goal is to simultaneously genotype TCRs and measure the mRNA expression of all genes. Integration of these data may lead to a better understanding of how TCR sequences correlate with the status and activity of T cells. More recently, the development of single cell technology had led to a rapid growth of transcriptome profiling of individual T cells [37–39]. Sequencing at the single cell level allows for straightforward pairing of TCR α and β chains, giving it an advantage over bulk mRNA sequencing. Another advantage of single-cell sequencing is that corresponding TCR alleles and expression signatures can be identified on a per-cell basis. This information could reveal new insights about interactions between TCRs and other genes that could otherwise be masked due to the mixture of cells in a bulk sample.

Although RNA-Seq is capable of revealing TCR sequences, its utility has not been addressed extensively. Previous studies mainly discussed technique requirements (e.g. the sequencing depth and read length) and algorithms that are suitable for TCR retrieval from RNA-Seq data [36, 38]. It remains unclear to what extent RNA-Seq can accurately reveal a TCR repertoire, given the proper sequencing protocols and algorithms applied. Despite the consensus that RNA-Seq may not be as powerful as targeted approaches due to lower coverage at the TCR loci, there is a lack of quantitative and systematic assessment of the use of RNA-seq for this purpose. Understanding the strengths and limitations of this technique is potentially important, especially for those with limited resources to employ single-cell methodologies. More information is needed about the minimum abundance of a TCR transcript that can be reliably detected by RNA-Seq, how accurately RNA-Seq can quantify the abundance of TCR transcripts, and also how well RNA-seq can characterize the distribution of TCR transcripts in a repertoire. These parameters may vary from bulk to single cell samples and may also depend on whether the repertoire is highly diverse or dominated by a few expanded clones. In single cell RNA-Seq, the number of cells sampled is another important factor that impacts the sensitivity. The choice of the sampling size likely relies on the diversity of the repertoire, the need to compensate for stochastic expression in individual cells, and the throughput of the technology used. Understanding these factors is critical for a comprehensive evaluation of RNA-Seq in TCR profiling. Ultimately, we would like to know under what circumstances RNA-seq data can be effective for characterizing TCR repertoire.

In this work, we use mouse as a model system to generate TCR repertoires of different diversity. The repertoire formed in the presence or absence of immunogenic challenge corresponds to a clonally expanded or non-expanded instance, respectively. Both the full-length single cell RNA-Seq and the bulk RNA-Seq are examined with respect to the above two types of repertoires. We are interested in the capability of RNA-Seq when its technique specifications meet the high standard of current practice. Thus, the samples are T-cell enriched, sequenced with high coverage, and with relatively long read length. We observe that the sensitivity of RNA-Seq may not adequately compare to what the targeted sequencing can offer. Nevertheless, given the sequencing depth and read length commonly used in practice, RNA-Seq in T-cell enriched samples can detect and quantify top TCR sequences whose frequency reaches several percentages or higher. Under circumstances where top enriched clones are of interest and transcriptome data is available, it is useful to conduct TCR analysis using the RNA-Seq data.

## Materials and methods

Regeneron Pharmaceutical Inc. is an AAALAC (Association for Assessment and Accreditation of Laboratory Animal Care) International-accredited research facility. All experiments involving laboratory animals, as described below, were carried out in accordance with the applicable regulations and standards as promulgated by the New York State Department of Health, Animal Welfare Act (USDA), PHS Policy (Office of Laboratory Animal Welfare), the ILAR Guide (Institute for Laboratory Animal Research) and Regeneron Pharmaceuticals, Inc. The protocols (Protocol #338 and Protocol #334), under which the experiments were conducted, were approved by Regeneron Pharmaceuticals, Inc. Institutional Animal Care and Use Committee (IACUC). All animal housing, husbandry and enrichment practices were in accordance with the Guide to the Care and Use of Laboratory Animals 8^th^ edition. All animals were monitored at least 2 to 3 times per week for body condition score and outward signs of distress, with include but are not limited to weight loss, lethargy, anorexia and dehydration. Tumor injections were operated under isoflurane anesthesia and mice were CO_2_-euthanized prior to tissue and tumor collection. All efforts were made to minimize suffering.

### Splenic T cells from LCMV (Lymphocytic Choriomeningitis Virus) mouse model

Wild type C57BL/6J mouse littermates either remained unchallenged or were injected intraperitoneally with 2x 10^5^ ffu of LCMV (lymphocytic choriomeningitis virus) Armstrong. Spleens were obtained from CO_2_-euthanized mice 7 days post infection. These procedures are described in approved IACUC Protocol #338. CD8^+^ T cells were isolated by immunomagnetic negative selection following manufacturer's instructions (StemCell Technologies #19853). CD8^+^ T cells were pelleted and stored at -20°C in Buffer RLT Plus from RNeasy Plus Mini Kit (Qiagen #74134).

### Tumor infiltrating and splenic T cells from MC38 tumor mouse model

Six-weeks old C57BL/6J mice were purchased from Jackson laboratory and maintained under pathogen-free conditions. MC38 murine colorectal adenocarcinoma cells were cultured in DMEM supplemented with 10% fetal bovine serum, Pen/strep, L-Glutamine, sodium pyruvate, HEPES and non-essential amino acid. One million MC38 cells were injected subcutaneously on each flank of a C57BL/6J mouse under isoflurane anesthesia to minimize discomfort. Tumor sizes were measured twice per week using calipers (Roboz RS-6466). Volume was calculated using the formula W^2^ × L × 0.5, where L is the longest dimension and W is the perpendicular dimension. Mice with tumors larger than 2000mm^3^ or with ulcerated tumors were euthanized by CO_2_ 14 days post-implantation before tumors and spleen were harvested. These procedures are described in approved IACUC Protocol #334.

Tumors and spleens were processed using Miltenyi gentleMACS Octo dissociator and mouse tumor dissociation kit (130-096-730) according to manufacturer’s instructions. Cells were stained with anti-CD8 antibody APC-eFluor 780 (eBioscience 47-0081-82) and DAPI (Sigma, D9564-10MG). CD8 positive live single cells were sorted by using Beckman Coulter MoFlo Astrios and subjected to Fluidigm C1 single cell sequencing.

### Single cell capture and RNA-Seq of CD8^+^ mouse T cells in MC38 tumor and spleen

Single T cells were suspended in C1 Cell Suspension Reagent (Fluidigm) and loaded onto 5- to 10-um-diameter 96-well C1 Integrated Fluidic Circuit (IFC, Fluidigm). Cells were subject to LIVE/DEAD staining (Fluidigm) and examined under a Nikon microscope for doublets and viability. Cells were subsequently lysed, and the mRNA was reverse transcribed and PCR amplified using C1 Single-cell Auto Prep IFC (protocol 100–7168 E1). The sequencing libraries were constructed via Nextera XT DNA Sample Prep Kit (Illumina). Sequencing was executed on Illumina HiSeq2500 by multiplexed single read run with 75 cycles (i.e. 1x75 bp reads). Raw BCL files were demultiplexed and converted to FASTQ via Illumina Casava 1.8.2. The data quality was confirmed by the FastQC analysis. No read trimming was applied prior to the downstream analyses. Reads in each cell were mapped to GRCm38/mm10 genome using ArrayStudio (Omicsoft) with one mismatch permitted. Cells were retained if they met the following criteria: they were positive for T cell marker genes (e.g. Cd3d/g/e), the total read count was equal or greater than 1 million, the ratio of uniquely mapped exon reads was equal or greater than 0.5, and the percentage of reads mapped to mitochondrial genes was no more than 0.25. Sequencing data from 92 tumor infiltrating T cells and 100 splenic T cells passed the initial quality test and were moved forward to the subsequent TCR analysis and gene expression profiling.

To derive differentially expressed genes between two groups of T cells, we first flagged genes in each cell as “absent” and “present” using an empirical minimum RPKM (reads per kilobases per million total reads) of 1. We excluded genes that were not flagged as “presence” in at least 50% of the cells of the higher expressing group. The fold change was computed as the ratio between the mean RPKM values of the two groups. The statistical significance (p-value) of the differential expressions were assessed by Student’s t-tests on the log-transformed RPKM values. We selected genes with fold changes no less than 1.5 in either the up or down directions with a p-value of at least 0.05 as the significantly perturbed gene signatures.

In parallel with the single cell RNA-Seq, the same pool of T cells was also sequenced without single cell capture. Typically, an aliquot containing ~2000–3000 cells was taken from the cell pool used for the single cell capture and sequenced as a whole by bulk RNA-Seq. SMARTer Ultra Low Input RNA Kit version 3 (Clontech) was applied for cDNA synthesis. Nextera XT DNA Sample Prep Kit (Illumina) was used to prepare RNA-Seq libraries. Sequencing was conducted on Illumina HiSeq2500 (Illumina) using multiplexed single read run with 80 cycles (i.e. 1x80bp reads). The raw data were reformatted as FASTQ using Illumina Casava 1.8.2. The full-length reads were of sufficient quality (FastQC) to be used as input for the TCR analysis programs. The sequencing FASTQ files are available from the European Nucleotide Archive database under accession number PRJEB27250.

### RNA-Seq of bulk CD8^+^ splenic T cells from naive and LCMV-infected mice

Total mRNA was extracted (MagMAX kit, Life Tech) and purified (Dyna beads mRNA Purification Kit, Invitrogen) from bulk samples of CD8^+^ splenic T cells of the naive and LCMV infected mice. Strand-specific RNA-Seq libraries were prepared from 500 ng RNA using KAPA stranded mRNA-Seq Kit (KAPA Biosystems). The libraries were amplified by twelve-cycle PCR. Sequencing was performed on Illumina HiSeq 2000 by multiplexed paired-read runs with 100 cycles (i.e. 2x100bp reads) at a deep coverage (110–150 million reads). The resulting FASTQ files were analyzed via FastQC to ensure sufficient data quality. There was no read trimming prior to the TCR analysis.

### 5’ RACE targeted sequencing of CD8^+^ splenic T cells from naive and LCMV-infected mice

Total RNA from sorted CD8^+^ splenic T cells were isolated from the cells using the Mag/Max-96 Total RNA Isolation kit (Thermo Fisher Scientific) according to manufacturer’s instructions. Reverse transcription was performed to generate cDNA containing TCRα or TCRβ constant region sequence, using a SMARTer RACE cDNA Amplification Kit (Clontech) with TCRα or TCRβ specific reverse-transcription primers. Purified TCR cDNAs were then amplified by 1st round PCR (semi-nested) and the resulting PCR products between 450-700bp were isolated using Pippin Prep (SAGE Science). These products were further amplified by a 2nd round PCR to attach Illumina adaptors and the sample index. Final PCR products between 400bp-700bp were isolated, purified, and quantified by qPCR using a KAPA Library Quantification Kit (KAPA Biosystems) before loading onto a Miseq sequencer (Illumina) for sequencing using Miseq Reagent Kits v3 (600 cycles). The Illumina sequences (2x301bp reads) were sorted based on the sample index perfect match and trimmed for quality. The median read length post trimming was 296bp. As a part of the QC procedure, overlapping pairs of trimmed reads were merged and the ones corresponding to rearranged TCRα or TCRβ chains were retained, based on the alignment to a mouse V and J segment database (IMGT GENE-DB, v3.1.18) using a local installation of IgBlast (NCBI, v2.2.25+). The retained sequences were analyzed by TCRklass [40] and MiTCR [36] with their default parameters for the targeted sequencing data. The overlaps of the TCR predictions were taken as the consensus results. The sequencing FASTQ files are available from the European Nucleotide Archive database under accession number PRJEB27272.

### Application of RNA-Seq based TCR typing programs

Computational programs such as TraCer [37] and MiTCR with parameters tailored for RNA-Seq [30, 41] were applied. All programs were provided with the mouse V and J allele sequences taken from IMGT GENE-DB (v3.1.18). Although reads were mapped to the V and J loci at the allele level, multiple programs (MiTCR, TCRklass) reported the final V and J typing at the gene level. To be consistent across the programs, the V and J predictions were consolidated at the gene level. In this work, a complete TCR was defined by the identity of the V and J genes together with the CDR3 sequence, also referred as V-CDR3-J in the text. The parameters used to execute the aforementioned programs can be found in S1 Supporting Information. In this study, we only focused on in-frame/productive V-CDR3-J recombination.

The prediction programs occasionally reported multiple homologous V genes to pair with the identical CDR3 & J gene counterparts when the supporting reads had non-unique V annotations. When a read was annotated with *N* ambiguous V genes, it contributed 1/*N* of a read to the abundance of each of the *N* TCRs. The reads with ambiguous TRBV annotations were insignificant. The rate was 0.3% in the bulk RNA-Seq data using the 100bp paired-end reads, 0.6% in the RNA-Seq of cell pools (80bp single-end reads) and 1% in the single cell RNA-Seq (75bp single-end reads). The mouse TRAV loci contain multiple gene duplication and triplication events, resulting in a higher sequence homology among TRAV genes, compared to the TRBV genes (Fig A in S1 Supporting Information). There are also seventeen pairs of TRAV genes, e.g. *TRAV14-1* and *TRAV14N-1*, sharing identical sequences. The rates of the reads with ambiguous TRAV annotations were 12%, 23% and 25% when the 100bp paired-end reads, 80bp, and 75bp single-end reads were employed, respectively. Note that the reads annotated with multiple TRAV genes identical in the sequence were not considered as ambiguous.

The non-uniquely annotated reads were kept in the TCR analyses for two reasons. First, not all the analysis programs used allow users to drop these reads (only TCRklass provides such an option). Second, we preferred to present all the information available in the data as is. We examined the consistency between the TCR transcript abundances measured with and without the non-uniquely annotated reads (Fig B in S1 Supporting Information). High correlation coefficients (0.87–0.99 for TCRα and > = 0.99 for TCRβ) were observed in all the datasets. As the overall TCR distribution remained consistent regardless of the ambiguous reads, a significant sampling bias might not be expected. Nonetheless, it was safer to keep these reads and report the ambiguous V predictions as is. Our efforts to control the ambiguity were in line with the capability of the current tools. For example, we executed the MiTCR program with parameters that have been optimized for the read lengths used in the study [30]. We also applied the module in TCRklass to assemble the overlapping pairs of reads into longer reads when applicable. The ambiguity may be fully resolved in the future by increasing the read length in the RNA-Seq practice or improving the prediction programs, which is out of the scope of the current study.

To ensure the quality of the results, consensus TCR predictions, defined as the overlap of at least two programs, were used in the subsequent analyses. An overlap TCR required identical V, CDR3 and J sequences. The rule was also applicable to TCRs with multiple V gene predictions. Besides matching the CDR3 and J gene identities, the V genes were intersected among multiple programs and only the overlapping subset, if any, was retained. Taking the consensus from multiple programs also helped reduce the TRAV ambiguity.

## Results

The power of the single cell and the bulk RNA-Seq in TCR profiling was assessed using repertoires with and without significant clonal expansion. For the single cell RNA-Seq, we chose T cells that infiltrate the *in vivo* implanted MC38 tumor as an example of a clonally expanded repertoire. T cells in the spleen of the tumor-bearing mice represent a highly diverse, non-expanded counterpart. The results were cross-validated with the RNA-Seq of the cell pools used in the single cell capture. Similarly, because the acute lymphocytic choriomeningitis virus (LCMV) infection is known to induce a potent antigen-specific expansion [5], we evaluated the bulk RNA-Seq using T cells retrieved from the LCMV-challenged mice versus those from naïve mice as the expanded and non-expanded repertoires, respectively. 5’RACE targeted TCR amplicon sequencing was applied to the same bulk samples for comparison. To ensure reliable TCR typing, we obtained consensus sequences from multiple available programs for all analyses.

### TCR profiling of mouse T cells by single cell RNA-Seq

The consensus TCR (V-CDR3-J) sequences were derived from whole transcriptome sequencing data from 92 MC38 tumor-infiltrating T-cells and 100 spleen T-cells, using several computer programs as described in Materials and Methods (see also S1 Supplementary Data). The number of cells examined was consistent with the typical throughput (10^2^ cells) of the Fluidigm C1 system. The proportion of reads that were mapped to the TCRα locus is 0.14–0.16%, and that to TCRβ is 0.18–0.23%. Detailed information about the read length, the coverage, and the mapping rates can be found in Table A in S1 Supporting Information. The differences between the results of the computer programs were minor (Fig C and D in S1 Supporting Information). The percentage of cells with TCRβ calls was higher than that with TCRα calls (79–85% vs 59–69%), which was consistent between the tumor and spleen control (Table 1). The lower detection rate of mouse TCRα is presumably attributed to the over 2-fold lower CDR3-containing reads covering the TCRα locus: the proportion of reads which were used for the CDR3 call is 10^−5^ for TCRα and 2–2.6x10^-5^ for TCRβ (Table A in S1 Supporting Information).

**Table 1:** Summary of the TCR detection by Fluidigm single cell RNA-Seq.

Tissue source	#T cells sequenced	Locus	#cells with TCR called	#cells with single TCRα (or TCRβ)	#cells with biallelic TCRα (or TCRβ)
MC38 tumor	92	TCRα	63 (69%)	56 (89%)	7 (11%)
		TCRβ	78 (85%)	77 (99%)	1 (1%)
Spleen	103	TCRα	59 (57%)	56 (95%)	3 (5%)
		TCRβ	79 (77%)	75 (95%)	4 (5%)

Most cells we sequenced contained a single TCRα and TCRβ, consistent with the known allelic exclusion in T lymphocytes. However, biallelic T-cell receptor rearrangement is also possible as a result of positive selection to enhance T-cell development [42]. We observed a small proportion of cells (1–11%) with two TCRα or two TCRβ alleles (Table 1), comparable to previous reports [37]. Microscopic examination of these cells revealed no sign of more than one cell in the well, excluding the possibility of the experimental artifact. The highest biallelic rate (11%) in TCRα in the MC38 tumor was unlikely to be caused by an artifact either, as 6 out of 7 cells with biallelic alleles share the identical clonotype sequences. The probability that all 6 of these cells would share the same clonotype due to random collision of two or more T-cells in the repertoire was very small.

For the cross-validation purpose, we also performed the transcriptome sequencing of the aliquots of the cell pools used for the single cell capture. Unlike the single cell approach, the bulk RNA-Seq of cell pools characterized the TCR repertoire at the transcript level instead of at the cell clone level. Nevertheless, it sampled the repertoire more thoroughly with a deep coverage of ~150 million reads (Table A in S1 Supporting Information). TCR sequences inferred from the cell pool sequencing (S2 Supplementary Data, Table A in S1 Supporting Information) were compared with the results derived from single cells.

**Fig 1:**
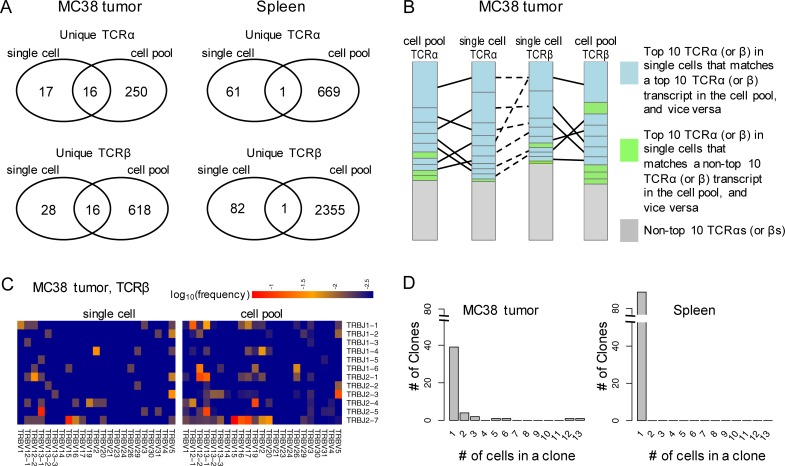
TCR profiling using the full length single cell RNA-Seq data. A) Unique TCRα and TCRβ sequences detected in the CD8+ T cells from the MC38 tumor and the spleen by single cell RNA-Seq, in comparison to the RNA-Seq of the aliquot of cell pools used for the single cell capture; B) Consistency of TCRα and TCRβ sequences detected in the MC38 tumor and in the spleen by RNA-Seq of single cells and the aliquot of cell pools used for the single cell capture. Each block represents one TCRα (or TCRβ) sequence with the height of the block proportional to its clonal abundance (based on the cell counts) in the single cell data or the transcript abundance (based on the read counts) in the cell pool data, respectively. Matches between the top 10 abundant TCRα (or TCRβ) sequences detected by either approach are connected by solid lines and the associated sequences are colored blue. Sequences detected by both approaches but ranked within the top 10 by only one are colored green. In the single cell dataset, the TCRα and TCRβ sequences observed in the same cell are connected with a dashed line. One TCRβ can be connected to two TCRαs (or vice versa) in cells with a biallelic TCRα (or TCRβ) locus. C) Usage of the TRBV and TRBJ genes in the MC38 tumor infiltrating T cells, measured in the single cells (left panel) and in the corresponding cell pool (right panel). The union of the TRBV (and TRBJ) genes detected in the two approaches are presented. D) Distribution of TCR clones in the MC38 tumor and the spleen detected by the single cell RNA-Seq. A clone refers to the unique pairing of TCRα and TCRβ sequences.

Fig 1A left panel and Table 2 indicates that, in the MC38 tumor, the majority of the TCRα and TCRβ sequences identified in the single cells and in the cell pool are consistent. For instance, there were 16 unique TCRα sequences observed in both datasets. The overlapping TCRα sequences corresponded to 47 cells (Table 2, S1 Supplementary Data), out of the total 63 T-cells whose TCRα sequences were detected (Table 1, S2 Supplementary Data). They also accounted for 73.2% of the total TCRα transcripts in the cell pool, according to the percentage of reads mapped to them (Table 2). Similarly, 16 unique TCRβ sequences, from 51 out of 78 cells, were confirmed in the RNA-Seq of the cell pool aliquots (Tables 1 and 2). This overlap enclosed 70.8% of the transcript reads in the cell pool (Table 2). Note that in the single cell data, the number of unique TCRα sequences (and TCRβ sequences) does not necessarily equal to the number of cells (Table 2), because multiple cells can share the same TCRα (and TCRβ) if they are from the same clone, and a cell with biallelic rearrangements can contain two different TCRα sequences (and/or TCRβ sequences).

**Table 2:** Comparison between the TCRs detected by the single cell RNA-Seq and the bulk RNA-Seq of the CD8+ T cells from the MC38 tumor and the mouse spleen.

Tissue source	Locus	measurement	single cell only	overlap	cell pool only
MC38 tumor	TCRα	#unique TCRs	17	16	250
		#cells	16 (1 biallelic)^a^	47 (6 biallelic)	⎯
		%reads	⎯	73.2%	26.8%
	TCRβ	#unique TCRs	28	16	618
		#cells	27 (1 biallelic)	51	⎯
		%reads	⎯	70.8%	29.2%
Spleen	TCRα	#unique TCRs	61	1	669
		#cells	58 (3 biallelic)	1	⎯
		%reads	⎯	1.3%	98.7%
	TCRβ	#unique TCRs	82	1	2355
		#cells	78 (4 biallelic)	1	⎯
		%reads	⎯	0.03%	99.97%

^a^ One cell among the 16 cells is biallelic at the TCRα locus. I.e. It has two unique TCRαs. The annotation is applicable to other similar expressions.

However, in the spleen where a non-expanded repertoire was expected (Fig 1A, right panel), only 1 out of 62 unique TCRαs detected in the single cells was observed in the cell pool. The shared TCRα sequence came from one cell and corresponded to 1.3% of the reads in the cell pool (Table 2). Similarly, only one TCRβ sequence was detected by both approaches. The number of cells (1) and the percentage of reads (0.03%) associated with this overlap were also low (Table 2). In contrast to the observations in the MC38 tumor, the limited overlap with the cell pool data suggests that the repertoire in the spleen may not be adequately depicted by the collection of single cells, although a comparable number of cells were profiled for both repertoires (Table 1). This finding illustrates an important factor to consider when applying the single cell RNA-Seq: how sufficiently a repertoire can be characterized depends on the diversity of the repertoire.

We next compared the major TCRs measured by single cell profiling with those in the cell pool aliquots of the MC38 tumor infiltrating T cells. As the TCRα and TCRβ sequences were not paired in the bulk cell pool samples, their abundances (i.e. frequencies) were calculated independently (S1 Supplementary Data). To be consistent, the abundances of the TCRα and TCRβ in the single cells were also calculated separately (S2 Supplementary Data). Fig 1B cross-compares the top 10 TCRα and TCRβ sequences detected in the single cells and in the cell pool data. The ranking is based on the clonal abundance or the transcript abundance in the single cell data or the cell pool data, respectively. It is evident that the majority of the top 10 abundant TCRα (and TCRβ) sequences in single cells are also among the top 10 in the cell pool, and vice versa. The good concordance validates the top enriched TCR clones identified by the single cell RNA-Seq. It also suggests that the clonal abundance of the major TCRs in this repertoire contributes to their transcript abundance.

In the T cells infiltrating the MC38 tumor, the V and J gene usage estimated by the single cell data and the cell pool data agreed well. As shown in Fig 1C, most of the enriched V-J pairs in the TCRβ locus are common between both profiling methods. For instance, *TRBV13-1*:*TRBJ2-4*, *TRBV15*/*TRBV16*/*TRBV17*/*TRBV20*:*TRBJ2-7*, *TRBV19*:*TRBJ2-4*, *TRBV5*:*TRBJ2-3*, *TRBV12-2*:*TRBJ2-1*, *TRBV2*:*TRBJ1-4*, *TRBV12-2*:*TRBJ1-6*, *TRBV13-1*:*TRBJ2-1* and *TRBV12-1*:*TRBJ1-1*. This result was expected because most of the TCRs from the single cells and the cell pool agreed on the full sequence constituted of V and J genes (Fig 1A left panel and Table 2). On the other hand, the abundance of a V-J combination was estimated differently in the single cells and the cell pool, referring to the percentage of cells and the percentage of reads associated with a V-J pair, respectively. A quantitative match between them may not be expected. In addition, the detection power in either approach may not be ideal given the available cell numbers or the sequencing coverage. Therefore, we also observed some non-overlapping V-J pairs (e.g. *TRBV13-1*:*TRBJ1-1*, *TRBV2*:*TRBJ2-7* and *TRBV5*:*TRBJ2-1*). Similar observations held at the TCRα locus (Fig E in S1 Supporting Information). Since there was little overlap between the TCRs identified by the single cell sequencing and RNASeq of cell pool in the spleen, it was difficult to obtain useful information therein.

Fig 1D summarizes the distribution of the clonotypes in the tumor and the spleen based on the single cell data. In the tumor, the top two clonotypes appeared in 13 and 12 cells, roughly 14.6% and 13.5% of the total 89 T cells in the MC38 tumor with TCRα and/or TCRβ detected (S1 Supplementary Data). The third to the tenth ranked clonotypes appeared in 6, 5, 3, 3, 2, 2, 2 and 2 cells, corresponding to a clonal abundance of 6.7%, 5.6%, 3.4%, 3.4%, 2.2%, 2.2%, 2.2% and 2.2%, respectively. The presence of highly abundant clones in the MC38 tumor confirmed clonal expansion of the TCR repertoire. In contrast, in the spleen, all clones appeared only once. The accompanying cell pool data for spleen also showed the abundance (i.e. frequency) of the most enriched TCR transcript as merely 0.1% (S2 Supplementary Data). The lack of abundant clones was consistent with the non-expanded repertoire in the spleen.

Our data help estimate the sensitivity of single-cell RNA-Seq for detecting TCR repertoires. The collection of ~100 cells matched with the standard throughput of the Fluidigm C1 platform. With this sampling capacity, TCR clones with 3% or higher frequencies (top 10 in S2 Supplementary Data) could be faithfully detected. In order to detect less abundant clones, it is necessary to increase the number of cells. In fact, significantly more cells may be needed to sample clones that are less enriched than those in our top 10. For instance, in our MC38 tumor sample with substantial clonal expansion, we estimated that 1000 cells may be needed to sample each of the top 30 clones (frequencies > = 0.2%) at least once (Fig F and associated text in S1 Supporting Information). Because the detection power depends on the repertoire diversity, a less expanded repertoire will require even more cells to reach a reasonable sensitivity. In the non-challenged spleen, we estimated that only the top 5 clones may be sampled using 1000 cells (Fig F, S1 Supporting Information). In the newer platforms for single cell RNA-Seq profiling, such as DropSeq and 10x genomics [43], 10^3^–10^4^ cells become feasible for fully interrogating clonal diversity. This large number of cells may help reduce the sampling hurdle discussed here.

Because the key advantage of using RNA-Seq data to characterize TCR repertoires is to simultaneously obtain and correlate information for both the TCR types and the genome-wide mRNA expression profiles, we examined the significantly perturbed genes in the top (the 13- and 12-cell) T cell clones in the MC38 tumor. We conducted four differential gene expression analyses (Materials and Methods). One comparison was the 13 T cells in the top clone in the MC38 tumor versus all spleen T cells. The other was the 12 T cells in the second-ranked clone in the MC38 tumor versus all spleen T cells. The third and fourth were the 13, and 12 T cells versus the singleton T cells in the MC38 tumor, respectively. Note the singletons T cells in the tumor may not be necessarily non-expanding due to the limited sampling. They may also originate from expanded clones but to a less extent. Using the singletons as additional negative controls allow us to focus on the gene perturbation most specific to the top expanded clones. We consider the 67 overlapping genes among the four comparisons as the specific signatures of the top clones infiltrating the tumor (see also Table B and Fig G in S1 Supporting Information).

**Fig 2:**
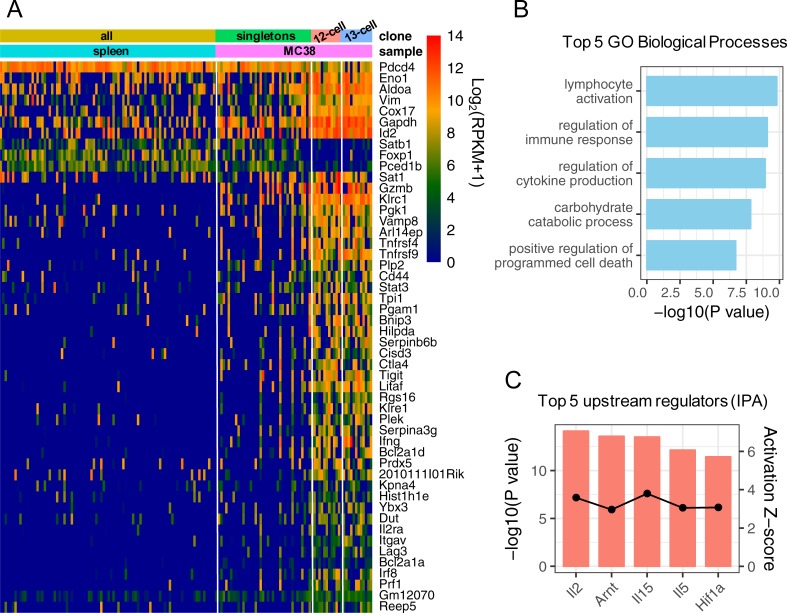
Gene signatures of most expanded T cell clones. A) top 50 (ranked by the fold change) out of the total 67 perturbed genes in the most expanded (12- and 13-cell) clones in the MC38 tumor. B) Gene Ontology Biological Processes that are significantly enriched in the perturbed genes in the most expanded clones. C) Significant upstream regulators of the perturbed genes in the most expanded clones identified by the Upstream Regulator Analysis in IPA.

Fig 2A shows the expression of the top 50 out of the 67 genes, ranked by the average fold change over the four comparisons. Gene Ontology analysis (Fig 2B) indicated that these genes were enriched for lymphocyte activation (e.g. *Gzmb*, *Prf1*), cytokine production (e.g. *Ifng*, *Irf8*, *Il2ra*) and programmed cell death (e.g. *Ctl4a*, *Tigit*, *Lag3*). The enrichment in lymphocyte activation and cytokine production genes suggested that the top clones were effector CD8^+^ T cells, which was consistent with the fact that they were expanded in the tumor, presumably upon tumor neo-antigen exposure. On the other hand, up-regulation of exhaustion markers was also expected for tumor-infiltrating effector T cells after prolonged antigen stimulation in the tumor microenvironment. The signature also directly supported the expansion of the top clones. For instance, *Tnfrsf9* and *Tnfrsf4*, which have been reported to promote CD8+ T cell expansion [44, 45], expressed much higher in the 13-cell and 12-cell clones. We applied the Ingenuity Pathway Analysis (IPA) to reveal the upstream regulators of the perturbed genes [46]. Fig 2C shows the top 5 individual regulators, ranked by the overlap p-value that measures the significance of whether a given regulator can explain the observed gene expression changes [46]. All of them have a positive activation score, given the up-regulation of their downstream genes. Among them, *IL-2* and *IL-15* are known important mitogens for antigen-stimulated CD8^+^ T cells [47–49]. Overall, the transcriptome signatures of the most expanded T cell clones are biologically meaningful.

### TCR profiling of mouse T cells by RNA-Seq of bulk samples

The mRNA of the splenic T cells from the LCMV-infected and the naïve mice were sequenced with paired-end long reads at deep coverage (Materials and Methods, Fig H in S1 Supporting Information). We evaluated the bulk RNA-Seq data against the 5’RACE targeted amplicon sequencing (Materials and Methods), which is considered the gold standard for TCR profiling [23]. Both methods measured the TCR transcripts in the repertoire. In the bulk RNA-Seq, we observed similar percentages of the mapped reads, the reads aligned to TCR regions and the reads used for V-CDR3-J sequence inference (Table C in S1 Supporting Information), as observed for the single cell RNA-Seq (Table A in S1 Supporting Information). This result is expected as both are whole transcriptome wide sequencing. The coverage of TCRα was about 4-fold less than that of TCRβ, also consistent with what we observed in the single cell RNA-Seq.

**Fig 3:**
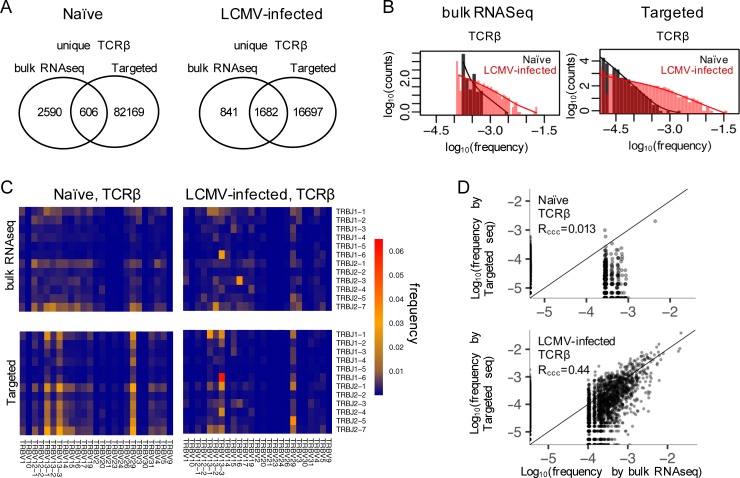
TCR profiling by bulk RNAseq. A) Unique TCRβ sequences detected in the non-challenged (naïve) and the LCMV-infected mouse splenic CD8^+^ T cells by bulk RNAseq, compared to the results obtained by the 5’ RACE targeted TCR sequencing. B) Abundance distribution of TCRβ sequences in the naïve and the LCMV-infected splenic T cells measured by the bulk RNAseq (left panel) or the targeted sequencing (right panel). The x-axis is the abundance (i.e. frequency) of each TCRβ and the y-axis is the number of TCRβ sequences at that abundance. C) Usage of the TRBV and TRBJ genes in the naïve and the LCMV-infected splenic T cells, measured by the bulk RNAseq (upper two panels) or the targeted sequencing (bottom two panels). The union of the TRBV (and TRBJ) genes detected in the two approaches are presented. D) Comparison of the quantitative estimation of the abundance (i.e. frequency) of TCRβ sequences that are commonly detected by both the bulk RNAseq and the targeted sequencing, under naïve or LCMV-challenged conditions.

5’ RACE targeted sequencing identified 18,379 and 82,775 unique TCRβ transcript sequences in the presence and absence of the LCMV infection, respectively (Fig 3A, right circles, see also Table C in S1 Supporting Information and S3 Supplementary Data). With the infection, fewer unique TCRβ sequences with infection indicated that the repertoire became less diverse. In addition, the infected repertoire had noticeably more TCRβs with an abundance (i.e. frequency) higher than 0.001, compared to the unchallenged repertoire (Fig 3B, right panel, red histogram). These observations were consistent with a clonal expansion upon the LCMV infection. The clonal expansion also resulted in the enrichment of specific TRBV and TRBJ sequences compared to the unchallenged condition (Fig 3C, bottom left & right panels). For instance, *TRBV13-3*:*TRBJ1-6* is the most abundant V-J combination, accounting for 6.5% of all observed V-J pairs observed. *TRBV13-1*:*TRBJ1-1* and *TRBV29*:*TRBJ2-5* follow. *TRBJ1-1*, *TRBJ2-1*, *TRBJ2-4* and *TRBJ2-7* also preferentially paired with *TRBV13-3*. *TRBV13-3* has been reported to be enriched in repertoires reactive to the gp33 antigen that is prevalently induced by the LCMV infection [50]. In addition, the most frequent TCRβ observed, *TRBV13-3*:CASSEGNYNSPLYF:*TRBJ1-6*, has been reported to be specific to the np396 antigen [50], another predominant antigen in the LCMV infection.

To assess the sensitivity of bulk RNA-Seq, we first looked at the non-expanded repertoire. 3196 unique TCRα sequences were identified by the bulk RNA-Seq in the absence of the LCMV challenge (Fig 3A, see also S4 Supplementary Data). 606 of them overlapped with the targeted sequencing. The overlap accounted for 20.1% of the total reads associated with the TCRβs in the bulk RNA-Seq, but only 2.3% of those in the targeted sequencing (Table 3, S3 Supplementary Data and S4 Supplementary Data). With the exception of the most enriched TCRα (*TRBV20*:CGARDRNTLYF:*TRBJ2-4*), the top ten sequences in the bulk RNA-Seq did not overlap with any other top ten sequences identified in the targeted sequencing of the non-expanded repertoire. In contrast, after the LCMV challenge, 1682 out of 2523 unique TCRβs detected by the bulk RNA-Seq overlapped with those by the 5’ RACE (Fig 3A). These overlapping TCRαs also captured the majority of the CDR3-containing reads in both the bulk RNA-Seq (89.8%) as well as in the targeted sequencing (70.5%) (Table 3). Six of the top ten TCRβs identified by each method were shared (S3 Supplementary Data and S4 Supplementary Data), including the np396-specific TCRβ discussed above. Similar conclusions held at the TCRα locus (Fig I, Table D in S1 Supporting Information, S3 Supplementary Data and S4 Supplementary Data).

**Table 3:** Comparison between the TCRβs detected by the bulk RNA-Seq and the targeted sequencing in the CD8+ T cells from the naïve and LCMV-challenged mice.

Tissue source	measurement	bulk RNA-Seq only	overlap	targeted Seq only
Naïve	#unique TCRβ	2590	606	82169
	%read (bulk RNA-Seq)	79.9%	20.1%	⎯
	%reads (targeted seq)	⎯	2.3%	97.7%
LCMV challenged	#unique TCRβ	841	1682	16697
	%reads (bulk RNA-Seq)	10.2%	89.8%	⎯
	%reads (targeted seq)	⎯	70.5%	29.5%

Overall the bulk RNA-Seq detected significantly fewer unique TCRβ sequences (Fig 3A) than the targeted sequencing. The bulk sequencing detected only 14% and 4% of unique TCRβs identified by the targeted sequencing under the LCMV-infected and the unchallenged conditions, respectively. In addition, the abundance distribution of the TCRβ sequences derived from the bulk RNA-Seq data (Fig 3B) had a narrower dynamic range and consequently a distorted distribution shape. This narrower range was expected given the substantially lower read coverage at the TCR loci. Regarding the V-J usage, the bulk RNA-Seq had a consistent enrichment of the combinations *TRBV13-3*:*TRBJ1-6* and *TRBV13-1*:*TRBJ1-1* V-J usage, consistent with targeted sequencing, but not in others such as *TRBV13-3*:*TRBJ1-1* and *TRBV29*:*TRBJ1-1* (Fig 3C, top panels). The magnitudes of the most enriched V-J pairs were less pronounced in the bulk sequencing as well. The results at the TCRα locus were similar. Compared to the targeted repertoire sequencing, the bulk RNA-Seq identified fewer TCRα sequences. The distribution of TCRα sequences and the V-J usages were skewed as well (Table D, Fig I, and Fig J in S1 Supporting Information, S3 Supplementary Data and S4 Supplementary Data). These observations suggest caution when applying bulk RNA-Seq to TCR repertoire profiling, especially for diverse repertoires. When the goal is to discover as many TCRs as possible in a repertoire, targeted sequencing may be the preferable sequencing choice.

Fig 3D illustrates the extent to which the bulk RNA-Seq could reliably quantify the abundance of the TCR transcripts. In the LCMV-infected condition, the concordance between the frequencies measured by the targeted sequencing and the bulk RNA-Seq is unsatisfactory for TCRαs with an abundance <1%. The naïve condition showed a lower concordance because of the more severe under-sampling when the repertoire diversity increased. We also observed similar issues at the TCRα locus (Fig I in S1 Supporting Information). This analysis suggests that bulk RNA-Seq may only be suitable for quantifying TCR transcripts whose abundance is over 1%. Consequently, the bulk RNA-Seq is more useful for characterizing an expanded repertoire in which many TCRs may be enriched above such a threshold, and is less useful for a non-expanded repertoire that contains few TCRs exceeding this threshold.

## Discussion

The main challenge in RNA-Seq-based TCR profiling is relatively limited sampling power. As shown in the current work, sequencing a bulk sample of T cells with ~ 150 million total reads may yield ~10^3^ CDR3-containing reads. These reads are far fewer than the number of cells (~10^7^) in a bulk sample, resulting in substantial under-sampling. The number of reads may drop by another order of magnitude or more in whole tissues or tumors, in which only a couple percentage of cells are T cells. Thus, it is not surprising to observe that the TCR identification and quantification in bulk RNA-Seq are most reliable for the top enriched transcripts. We estimate the abundance threshold for quantitative detections to be about 1% given the current standard of the read length and the coverage. Expanded TCRs in a repertoire usually exceed such a threshold, whereas those in a highly diverse repertoire rarely do. Consistently, we observed in this study that RNA-Seq faithfully captured the top enriched TCR transcripts from the expanded repertoires, but merely sampled random ones from the non-expanded repertoires. The ability to determine expanded TCRs is important. Knowing their TCR sequences and abundances can lead to an understanding of T cell specificity and eventually to the development of strategies to take advantage of the specificity. It is therefore worthwhile to perform TCR analysis on expanded repertoires using RNA-Seq data when transcriptome data already exist or are convenient to obtain.

On the other hand, there are circumstances in which measurement of less abundant TCR transcript sequences is critical. For instance, changes in the number of transcripts with low to intermediate abundance (0.001%-0.1%) may differentiate anti-PD1/anti-CTLA4 responders from non-responders during checkpoint inhibitor treatments [14, 51]. In these cases, bulk RNA-Seq may not be suitable due to limited ability to sample and quantify lower abundant transcripts. Under these circumstances, it would make more sense to employ targeted TCR profiling. The state-of-the-art targeted platforms (e.g. ImmunoSEQ, iRepertoire) can robustly type millions of T cells. Some of them can also couple the alpha and beta chains with extremely high accuracy (e.g. pairSEQ, AbPair).

Compared to the bulk RNA-Seq, the full-length single cell transcriptome sequencing using Fluidigm C1 platform has much higher TCR coverage (~10^2^ CDR3 containing reads per cell). Consequently, TCR sequences can be detected in the majority of the captured cells: ~80% in this work and similar or higher percentages in other reports [39]. In addition to pairing the alpha and beta chains, the unique advantage of single cell RNA-Seq is the simultaneous detection of genome-wide gene expression in clones of interest. The bottleneck is the ability to sample a large number of cells, contingent on the technology throughput and cost. The number of T cells harvested in this study was limited to the throughput of the Fluidigm C1 system. We chose the C1 platform because it allows sequencing of the full-length transcripts so that direct TCR typing from the data is possible. The typical throughput of the C1 system is in the order of hundreds of cells [52, 53]. Evaluating its capability in TCR profiling at the same scale can be useful. By assessing ~100 cells both in the tumor and in the spleen, we have observed dramatic differences in the TCR distribution that correspond to the expanded and non-expanded repertoires, respectively. The results suggest that it is possible to obtain useful information about the TCR repertoire via single cell RNA-Seq using the C1 platform. Similarly, in a recent study, perturbations in T cell repertoires induced by the Salmonella infection were detected based on the C1 single cell sequencing of ~50 cells [37]. Nevertheless, given the limited number of cells, the clonal abundance threshold for a reliable detection derived herein (~3%) may be only referred as an upper limit. It may be refined with a larger number of cells in the future.

It is intriguing that sampling 10^3^−10^4^ cells now becomes possible with promising new single cell platforms such as DropSeq and 10x genomics [54], potentially overcoming the hurdle of the sampling power. More excitingly, a few recently released platforms enable the whole transcriptome sequencing and the targeted TCR typing in the same cells and in a high throughput manner (e.g. Chromium Single Cell Immune Profiling by 10x Genomics Inc.). These new technologies may provide better methods for conducting TCR profiling and integrating it with the genome-wide gene expression. In the future, such integration may help identify new means to engage or abolish the effector T cells for therapeutic purposes.

## Supporting information

S1 Supporting Information Collection of Table A-D, Fig A-J and supplementary texts.
**Table A. RNA-Seq protocol and mapping statistics of the single CD8^+^ T cells and the accompanying cell pools in the mouse MC38 tumor and spleen**.
**Table B. 67 significantly perturbed genes in the top expanded T cell clones in the MC38 tumor**.
**Table C. RNA-Seq protocol and mapping statistics of the bulk RNA-Seq and the 5’ RACE targeted sequencing of CD8^+^ T cells from the naïve and LCMV-challenged mice**.
**Table D. Comparison between the TCRαs detected by the bulk RNA-Seq and the targeted sequencing in the CD8^+^ T cells from the naïve and LCMV-challenged mice**.
**Fig A. Percentage sequence identity among mouse TRAV and TRBV genes.** The sequence identity is defined as the percentage of matching nucleic acid bases between a pair of V genes. For multi-allele genes the best percentage of identity between their alleles is shown. Sequences are obtained from IMGT GENE-DB, v3.1.18.
**Fig B. Consistency between the transcript abundance (i.e. frequency) of the TCRα and the TCRβ measured with and without the non-uniquely annotated reads.** Data sources are the bulk RNA-Seq (1x80bp) of T cell pools from the mouse MC38 tumor and the spleen (A, C), and the bulk RNA-Seq (2x100bp) of splenic T cells in the naïve and LCMV-infected mice (B, D). The calculations are based on the outputs from TCRklass that offers an option to include or exclude the ambiguous reads. The Pearson correlation coefficients (R) are shown.
**Fig C. Derivation of consensus TCR sequences in single cell RNA-Seq of mouse CD8^+^ T cells from MC38 tumor and spleen**.
**Fig D. Derivation of consensus of TCR sequences using RNA-Seq of the aliquots of the CD8^+^ T cells used for the single cell capture from the mouse MC38 tumor and spleen**.
**Fig E. Usage of the TRAV and TRAJ genes in MC38 tumor infiltrating T cells**. The frequency of usage was measured by either the single cell RNAseq (left panel) or the bulk RNA-Seq of corresponding cell pools (right panel). The union of the TRAV (and TRAJ) genes detected in the two approaches is presented.
**Fig F. The impact of the cell numbers on the detection power of the single cell RNA-Seq**.
**Fig G. Significantly perturbed genes in the top expanded T cell clones in the MC38 tumor.** The specific signatures for the top expanded T cell clones infiltrating the tumor refer to the 67 overlapping genes among the following four comparisons: the most expanded (13-cell) clone versus the singleton clones in the MC38 tumor infiltrating T cells (I), the second most expanded (12-cell) clone versus the singleton clones in the MC38 tumor infiltrating T cells (II), the most expanded (13-cell) clone in the MC38 tumor infiltrating T cells versus all the clones in splenic T cells (III), and the second most expanded (12-cell) clone in the MC38 tumor infiltrating T cells versus all the clones in splenic T cells (IV).
**Fig H. Derivation of consensus of TCR sequences from targeted (5’ RACE) sequencing and from bulk RNA-Seq of CD8^+^ splenic T cells from naïve and LCMV-infected mice**.
**Fig I. Comparison of TCRα detection by the bulk RNA-Seq and the targeted sequencing**.
**Fig J. Comparison of the TRAV and TRAJ usages measured by the bulk RNA-Seq and the targeted sequencing in the naïve and LCMV-challenged splenic T cells**.
(PDF)

S1 Supplementary Data (XLSX)

S2 Supplementary Data (XLSX)

S3 Supplementary Data (XLSX)

S4 Supplementary Data (XLSX)

S1 Supplementary File (ZIP)
