# Supplementary material for: Evaluation of the capacities of mouse TCR profiling from short read RNA-seq data
Source: PLoS One. 2018 Nov 15;13(11):e0207020. doi: 10.1371/journal.pone.0207020 (PMC6237323; doi:10.1371/journal.pone.0207020)
Supplement: S1 Supporting Information — Collection of Table A-D, Fig A-J and supplementary texts. Table A. RNA-Seq protocol and mapping statistics of the single CD8+ T cells and the accompanying cell pools in the mouse MC38 tumor and spleen. Table B. 67 significantly perturbed genes in the top expanded T cell clones in the MC38 tumor. Table C. RNA-Seq protocol and mapping statistics of the bulk RNA-Seq and the 5’ RACE targeted sequencing of CD8+ T cells from the naïve and LCMV-challenged mice. Table D. Comparison between the TCRαs detected by the bulk RNA-Seq and the targeted sequencing in the CD8+ T cells from the naïve and LCMV-challenged mice. Fig A. Percentage sequence identity among mouse TRAV and TRBV genes. The sequence identity is defined as the percentage of matching nucleic acid bases between a pair of V genes. For multi-allele genes the best percentage of identity between their alleles is shown. Sequences are obtained from IMGT GENE-DB, v3.1.18. Fig B. Consistency between the transcript abundance (i.e. frequency) of the TCRα and the TCRβ measured with and without the non-uniquely annotated reads. Data sources are the bulk RNA-Seq (1x80bp) of T cell pools from the mouse MC38 tumor and the spleen (A, C), and the bulk RNA-Seq (2x100bp) of splenic T cells in the naïve and LCMV-infected mice (B, D). The calculations are based on the outputs from TCRklass that offers an option to include or exclude the ambiguous reads. The Pearson correlation coefficients (R) are shown. Fig C. Derivation of consensus TCR sequences in single cell RNA-Seq of mouse CD8+ T cells from MC38 tumor and spleen. Fig D. Derivation of consensus of TCR sequences using RNA-Seq of the aliquots of the CD8+ T cells used for the single cell capture from the mouse MC38 tumor and spleen. Fig E. Usage of the TRAV and TRAJ genes in MC38 tumor infiltrating T cells. The frequency of usage was measured by either the single cell RNAseq (left panel) or the bulk RNA-Seq of corresponding cell pools (right panel). The union of the TRAV (and TRAJ) [file pone.0207020.s001.pdf]

Table A

Tissue source	Sample type	Locus	sequencing protocol	sequencing depth(Million)	rate of total reads mapped	rate of reads mapped to TCR loci[a]	rate of reads used for CDR3 call[b]
MC38 tumor	single cell	TCRα	1x75bp	2.2 per cell	0.66	1.6E-03	1E-05
		TCRβ				1.8E-03	2.0E-05
	cell pool	TCRα	1x80bp	159.1	0.93	1.1E-03	2.6E-05
		TCRβ				3.1E-03	5.1E-05
Spleen	single cell	TCRα	1x75bp	2.6 per cell	0.64	1.4E-03	1E-05
		TCRβ				2.3E-03	2.6E-05
	cell pool	TCRα	1x80bp	128.6	0.87	1.0E-03	2.0E-05
		TCRβ				3.4E-03	5.2E-05

[a] The percentage is calculated as the mean ratio of the number of reads mapped to all the TCRV/J/D/C segments versus the total number of mapped reads

[b]The percentage is calculated as the mean ratio of the number of the high quality CDR3 containing reads to the total number of mapped reads over all available samples.

Table B

Symbol	fold change[a]	Symbol	fold change	Symbol	fold change
2010111I01Rik	34.6	*Vamp8*	3.9	*Igf2r*	2.8
Klre1	33.2	*Fkbp1a*	3.9	*Sept11*	2.8
Satb1	-30.9	*Prdx5*	3.8	*Gapdh*	2.7
Ifng	17.7	*Klrc1*	3.7	*Dut*	2.6
Pced1b	-11.8	*Pdcd4*	-3.6	*Cox7c*	2.6
Itgav	11.4	*Prf1*	3.6	*Id2*	2.6
Serpina3g	10.7	*Stat3*	3.5	*Pkm*	2.5
Bnip3	10.6	*Ybx3*	3.4	*Sat1*	2.4
Tnfrsf9	8.3	*Arl14ep*	3.3	*Gzmb*	2.4
Il2ra	8.3	*Cisd3*	3.2	*Ost4*	2.4
Serpinb6b	7.7	*Eno1*	3.2	*Gm12070*	2.4
Rgs16	7.0	*Cd44*	3.2	*Hprt*	2.3
Vim	7.0	*Tpi1*	3.2	*Rps27rt*	-2.3
Pgk1	6.7	*Gm5643*	3.2	*Eno1b*	2.2
Litaf	6.5	*Hist1h1e*	3.1	*Ctla4*	2.2
Lag3	6.0	*Prickle3*	3.1	*Tigit*	2.2
Bcl2a1d	5.4	*Irf8*	3.1	*Spcs3*	2.1
Bcl2a1a	5.3	*Foxp1*	-3.1	*Gm6682*	2.1
Kpna4	5.0	*Plp2*	3.0	*Higd1a*	2.1
Hilpda	4.7	*Reep5*	3.0	*Akr1a1*	2.1
Tnfrsf4	4.3	*Aldoa*	2.9	*Fxyd5*	2.0
Plek	4.1	*Pgam1*	2.9		
Cox17	4.1	*Rps27*	-2.8		

[a] Average fold change over the comparisons of the 13- and 12-cell clones to the singletons in the MC28 tumor and the spleen.

Table C

Tissue source	Sample type	Locus	sequencing protocol	sequencing depth (Million)	rate of reads mapped	rate of reads mapped to TCR[a]	rate of reads used for CDR3 derivation[b]
LCMV infected	bulk	TCRα	2x100bp	148.5	0.912	1.0E-03	4E-05
		TCRβ				3.8E-03	8.6E-05
	targeted	TCRα	2x301bp	0.32	0.91		0.60
		TCRβ		0.45	0.97		0.89
naïve	bulk	TCRα	2x100bp	108.7	0.905	1.5E-03	3.2E-05
		TCRβ				6.3E-03	6.6E-05
	targeted	TCRα	2x301bp	0.41	0.71		0.49
		TCRβ		0.47	0.96		0.68

[a] The percentage is calculated as the mean ratio of the number of reads mapped to all the TCRV/J/D/C segments versus the total number of mapped reads

[b]The percentage is calculated as the mean ratio of the number of the high quality CDR3 containing reads to the total number of mapped reads over all available samples.

Table D

Tissue source	measurement	bulk RNA-Seq only	overlap	targeted Seq only
Naïve	#unique TCRα	1307	182	18101
	%reads (bulk RNA-Seq)	85.0%	15.0%	–
	%reads (targeted seq)	–	3.2%	96.8%
LCMV challenged	#unique TCRα	554	750	11162
	%reads (bulk RNA-Seq)	22.2%	77.8%	–
	%reads (targeted seq)	–	54.0%	46.0%

Fig A

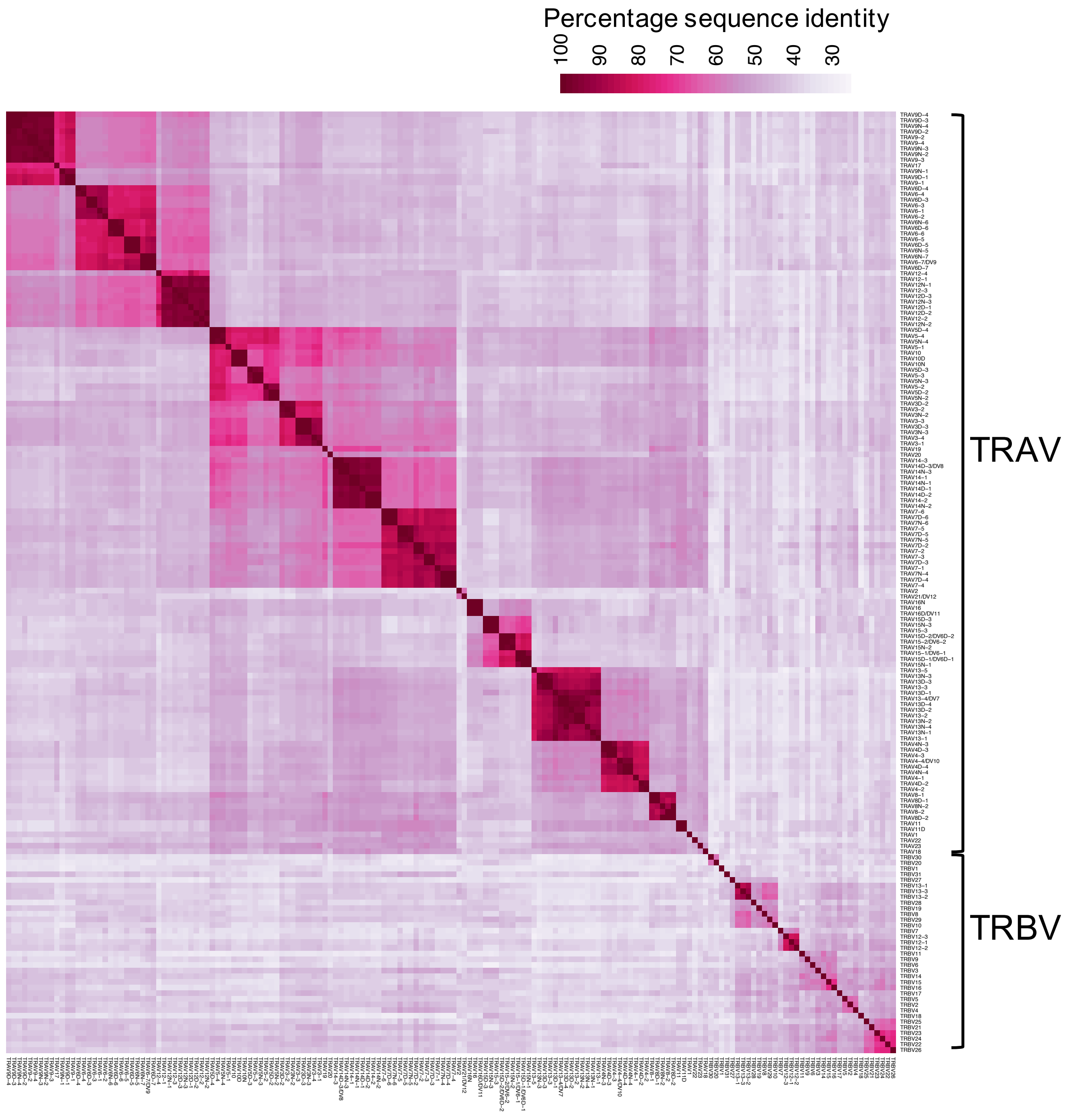

Percentage sequence identity
100
90
80
70
60
50
40
30
TRAV
TRBV

Fig B

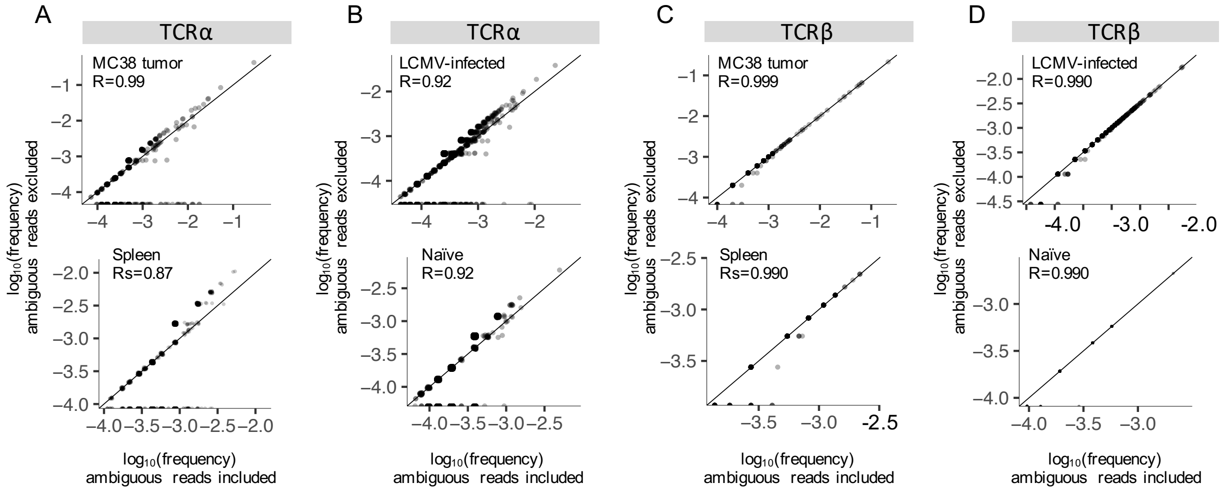

Fig C

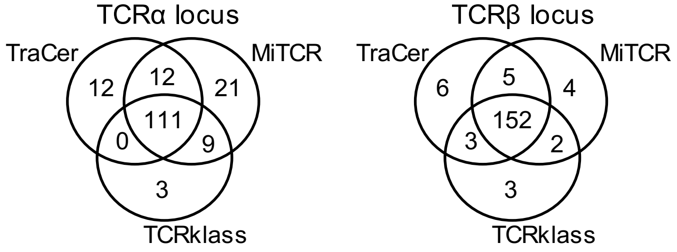

Fig D

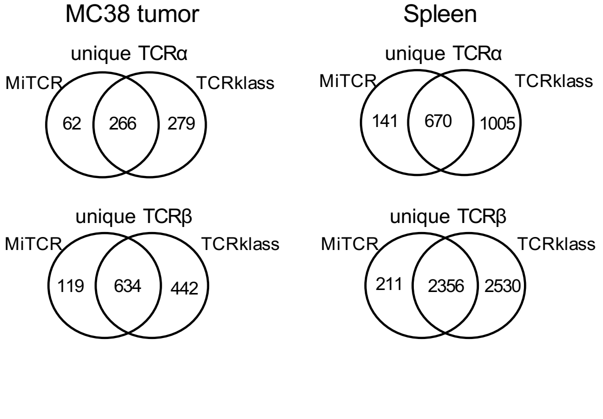

Fig E

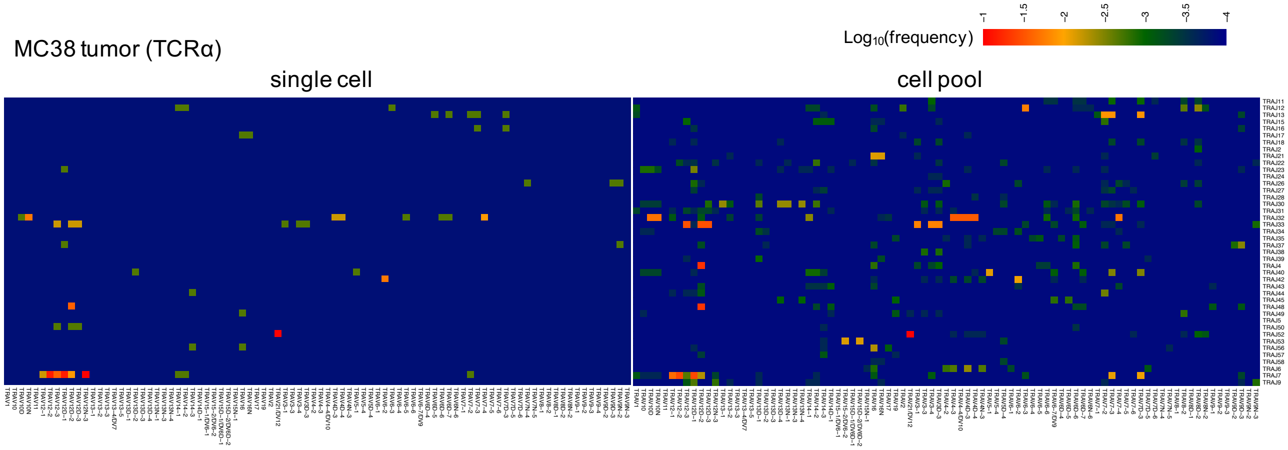
MC38 tumor (TCRα)
Log10(frequency)
-1
-1.5
-2
-2.5
-3
-3.5
-4
single cell
cell pool

Fig F

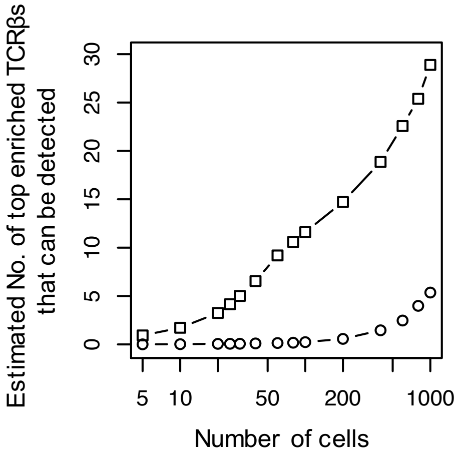
Estimated No. of top enriched TCRβs that can be detected
0
5
10
15
20
25
30
5
10
50
200
1000
Number of cells

Fig G

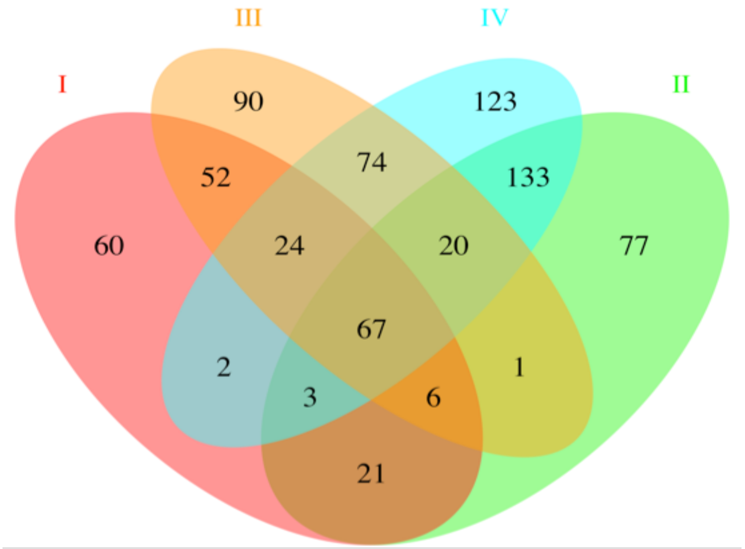

I: 13-cell clone vs. all singleton clones in MC38 tumor
II: 12-cell clone vs. all singleton clones in MC38 tumor
III: 13-cell clone in MC38 tumor vs. all clones in spleen
IV: 12-cell clone in MC38 tumor vs. all clones in spleen

Fig H

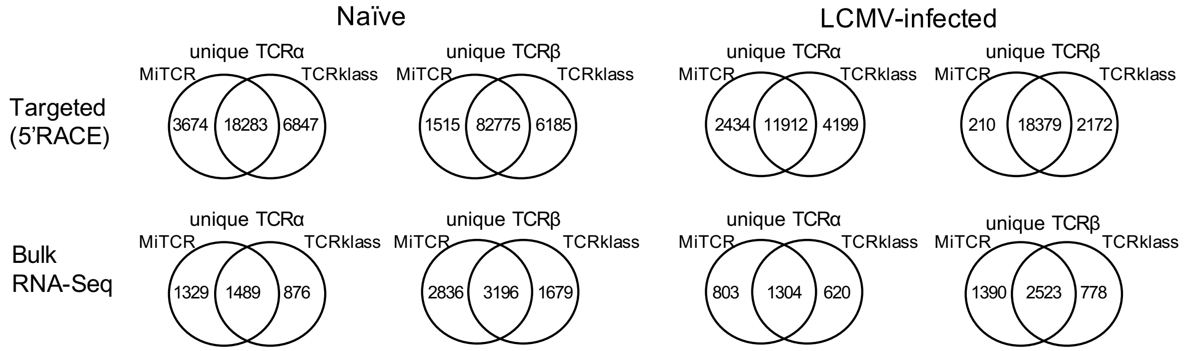

Fig I

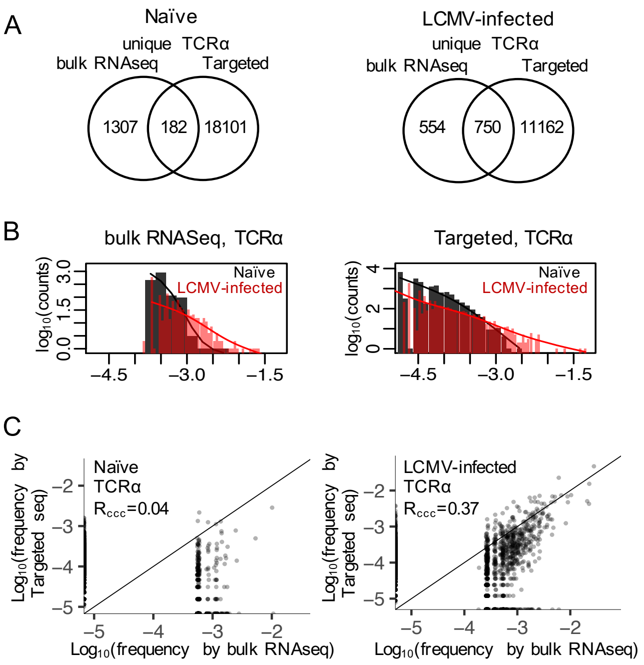
A
Naïve
unique TCRα
bulk RNAseq
Targeted
1307
182
18101
LCMV-infected
unique TCRα
bulk RNAseq
Targeted
554
750
11162
B
bulk RNASeq, TCRα
Targeted, TCRα
log10(counts)
Naïve
LCMV-infected
C
Naïve
TCRα
Rccc=0.04
LCMV-infected
TCRα
Rccc=0.37
Log10(frequency by Targeted seq)
Log10(frequency by bulk RNAseq)

Fig J

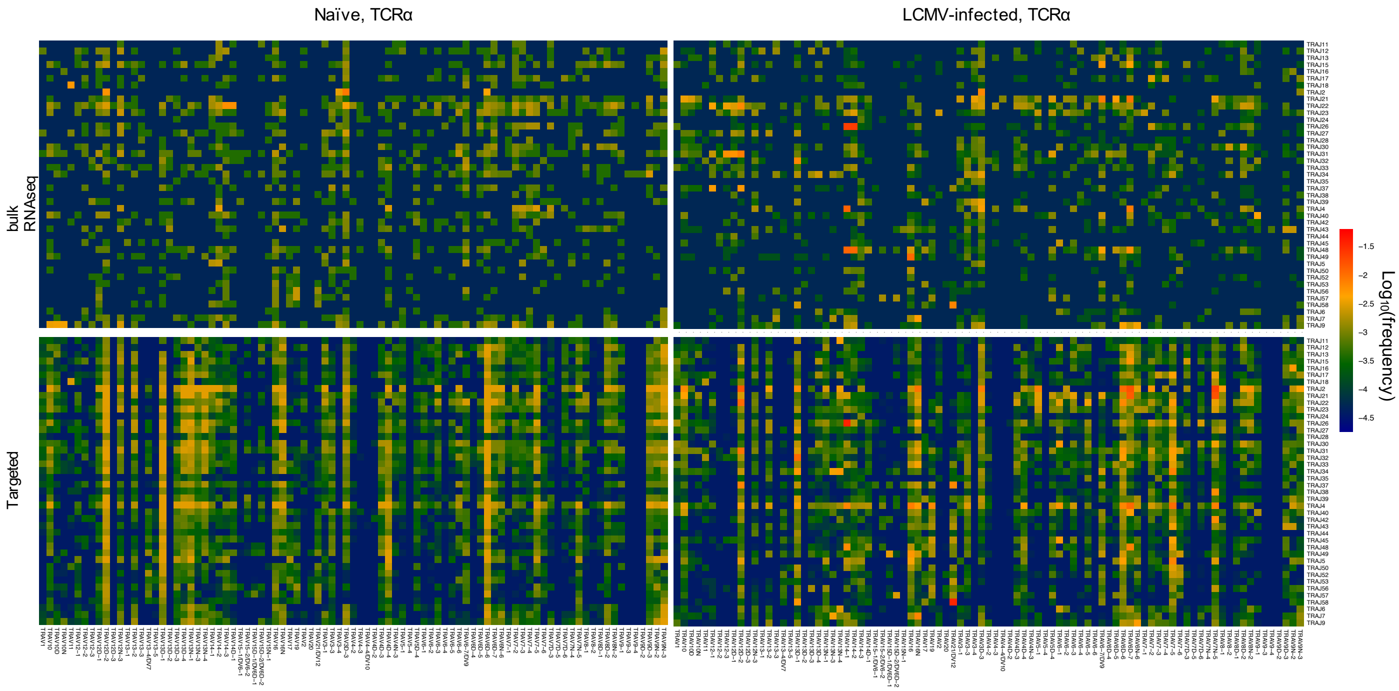
Naïve, TCRα
LCMV-infected, TCRα
bulk RNAseq
Targeted
Log10(frequency)

Parameters used in published programs for RNA-Seq based TCR profiling

When TraCer was applied to the single cell data, all parameters were set at default. When running MiTCR we adopted the parameters tailored for RNA-Seq in a previous study [1]. More specifically, the parameters for the single cell data (read length = 75bp) are: minAlignmentMatches = 18, 11, 12 and 18 for TRAV, TRAJ, TRBV and TRBJ, respectively and qualityInterpretationStrategy = 25. In case of the bulk RNA-Seq (read length = 100bp), the parameters are: minAlignmentMatches = 12, 19, 14 and 16 for TRAV, TRAJ, TRBV and TRBJ, respectively and qualityInterpretationStrategy = 35.

In order to use TCRklass on transcriptome data, we first mapped the reads to the mouse transcriptome (GRCm38/mm10) using TopHat2 (version 2.0.12). The reads aligned to any of the V, D, J, C regions of the TCRα (or the TCRβ) locus were saved for future usage. The remaining reads were mapped by Bowtie2 (version 2.2.3) against the collection of all the V and J allelic sequences in mouse (IMGT GENE-DB, v3.1.18), wherein both a global (--end-to-end, --very-sensitive) and a local alignment (--very-sensitive-local) were executed and the mapped reads were collected. In the end, all above collected reads were analyzed by TCRklass (0.6.0). The parameters used can be found in S6 Supplementary File.

Impact of cell numbers on the TCR detection power in single cell RNA-Seq

We estimated the sampling power of single cells in terms of how many clones in a repertoire may be detected given the number of cells. For this exercise, we focused on TCRβ for its higher coverage and typing consistency. We approximated the distribution of TCRs in the mouse MC38 tumor and spleen using the RNA-Seq data of the aliquots from their respective cell pools. The frequency of a TCRβ was calculated as the percentage of the reads mapped to it among all detected TCRβs (S3 Supplementary Data). The TCRβs were sorted in descending frequency. For instance, the 30th enriched TCRβ in this dataset corresponds to a frequency of ~0.2%. We then sampled the TCRβs in the MC38 tumor (or spleen) cell pool with probabilities proportional to their frequencies. For a given cell number *N*, we performed the sampling *N* times, and examined how many top frequent TCRβs (from top 1 counting downwards) were detected at least once. This value serves as an estimation of the detection power of *N* single cells. Fig F summarizes the detection power of 5-1000 cells, each averaged over 10 repeated samplings. Note that the TCR distribution in the cell pools was measured based on the transcript abundance, which may not reflect the actual clonal abundance in the repertoire. The results here may be considered at most an order-of-magnitude estimation.

1. Brown SD, Raeburn LA, Holt RA. Profiling tissue-resident T cell repertoires by RNA sequencing. Genome Med. 2015;7:125.
